# Transient minimal hydronephrosis on contralateral kidney in infants with unilateral hydronephrosis: Is it an early sign of worsening of the affected kidney?

**DOI:** 10.3906/sag-2012-99

**Published:** 2021-08-30

**Authors:** Mirzaman HUSEYNOV, Rahşan ÖZCAN, Şenol EMRE, Nur CANPOLAT, Sebuh KURUĞOĞLU, Haluk Burçak SAYMAN, Mehmet ELİÇEVİK, Yunus SÖYLET, Cenk BÜYÜKÜNAL, Haluk EMİR

**Affiliations:** 1 Department of Pediatric Surgery, Cerrahpaşa Medical Faculty, İstanbul University-Cerrahpaşa, İstanbul Turkey; 2 Division of Pediatric Nephrology, Department of Pediatrics, Cerrahpaşa Medical Faculty, İstanbul University-Cerrahpaşa, İstanbul Turkey; 3 Division of Pediatric Radiology, Department of Radiology, Cerrahpaşa Medical Faculty, İstanbul University-Cerrahpasa, İstanbul Turkey; 4 Department of Nuclear Medicine, Cerrahpasa Medical Faculty, İstanbul University-Cerrahpaşa, İstanbul Turkey; 5 Division of Pediatric Urology, Department of Pediatric Surgery, Cerrahpaşa Medical Faculty, İstanbul University-Cerrahpaşa, İstanbul Turkey

**Keywords:** Infant, compensatory hypertrophy, transient hydronephrosis, ureteropelvic junction obstruction

## Abstract

**Background/aim:**

The criteria for surgical management of ureteropelvic junction obstruction are not well-defined, and there is a risk for loss of renal function before the operation. In this context, certain changes in contralateral kidney had been investigated in order to increase the sensitivity of diagnosis. In this study, we aimed to investigate whether contralateral transient minimal hydronephrosis (CTMH) can be considered as an “early alarm” sign for worsening of the affected kidney in infants with hydronephrosis.

**Materials and methods:**

A total of 182 infants (92 surgically treated and 90 conservatively followed-up) with unilateral hydronephrosis were retrospectively analyzed. Ultrasonography and renal scan findings were evaluated. Correlation between the appearance of CTMH, contralateral compensatory hypertrophy (CCH) on ultrasonography, and prognosis of the affected kidney were evaluated.

**Results:**

Among the surgically treated patients, 18 (19.6%) patients developed CTMH on average 7 months (0–13 months) before surgery. Among these 18 patients with CTMH, 12 patients (66.6%) had loss of renal function preoperatively, while this ratio was 29.7% on their counterparts (p = 0049). CCH was observed in 31 (33.7%) individuals in surgically treated patient group including all 18 patients with CTMH, while none of the conservatively followed-up patients developed CCH and/or CTMH. In the multiple logistic regression analysis, among the variables investigated, CTMH was found as an independent predictor of the deterioration in the affected kidney and of the poor prognosis (p = 0.011 and p = 0.0004, respectively).

**Conclusion:**

In our study, among the variables investigated, CTMH was found as an independent predictor of the deterioration in the affected kidney and poor prognosis in infants followed-up with isolated unilateral hydronephrosis. Additionally, CTMH can be considered as an “early alarm” sign for worsening of the affected kidney and the need for surgical intervention.

## 1. Introduction

Hydronephrosis (HN) is the most common urinary tract pathology detected in the prenatal period. Some of the patients with prenatally diagnosed HN have ureteropelvic junction obstruction (UPJO) and need surgical intervention [1]. As none of the available investigations can make a definite diagnosis of urinary obstruction, differentiation between obstructive (UPJO) and non-obstructive HN is usually made according to clinical findings and anatomical or functional status of affected kidney [2–5]. In this context, changes in the contralateral kidney have also been investigated in order to increase the sensitivity of the diagnosis [6]. 

In this retrospective study, we described “contralateral transient minimal hydronephrosis” (CTMH) as a new clinical phenomenon and investigated its relation with functional changes in the affected kidney in unilateral hydronephrosis cases.

## 2. Materials and methods

After the approval of the ethics committee, medical records of patients below 1 year of age who were presented with HN between the years 2005 and 2016 were retrospectively reviewed. A total of 182 patients with isolated HN were included. According to treatment, the patients were divided into two groups as surgically-treated and conservatively follow-up patients. All clinical features including age, sex, age of presentation, age of operation, ultrasound examination of the affected and contralateral kidney, and scintigraphy findings were collected from patient files.

### 2.1. Determination of follow-up protocols, ultrasonographic (US), scintigraphic examinations 

All patients were evaluated by the Pediatric Nephro-Urology Council (Pediatric Urology, Nephrology, Radiology and Nuclear Medicine specialist) of our hospital. The imaging modalities and timing were discussed and decided individually.

For anatomical parameters, detailed US examinations were performed by the same pediatric radiology team, and the same parameters such as HN grade [SFU (Society of Fetal Ultrasound) grade], renal pelvis anterior-posterior diameter (RPAPD), parenchymal thickness, parenchymal echogenicity, corticomedullary differentiation, uroepithelial thickness had been evaluated and reported. None of the patients were hydrated intravenously before US examinations. 

The percentile values of the kidneys for evaluating CCH were calculated using Multivariable Pediatric Renal Nomogram based on the studies of Chen et al [7,8]. Contralateral compensatory hypertrophy was defined as the kidney length above the 95th percentile of normal length according to age, sex, and height. Minimal hydronephrosis was defined as SFU Grade I-II hydronephrosis (RPAPD ranged 5–10 mm) of the contralateral kidney. Minimal hydronephrosis during full bladder and/or which did not persist at the end of the examination was not selected as minimally HN. Contralateral transient minimally hydronephrosis was defined as a minimally HN, which was not present initially, developed during follow-up and regressed by time. Progressive hydronephrosis was defined as an increase in RPAPD and caliceal diameter, parenchymal echo deteriorations, and parenchymal thinning in consecutive ultrasonography examinations.

For functional status of the kidneys, Tc-99m DMSA or Tc-99m MAG-3 scintigraphy was performed in selected caseses depending on the severity of HN and clinical symptoms. Although scintigraphy selection used to be determined by the availability of the material and the age of the patient, MAG-3 was preferred in our clinic afterwards. Preoperative DRF < 40% was defined as a decreased renal function. Loss of renal function was defined as >10% loss of DRF in consecutive scintigraphy. At least 10% reduction in DRF and/or deterioration in HN following the operation was identified as postoperative poor outcome.

Except for emergency surgical/interventional indications such as severe HN presented with palpable abdominal mass or urinary infection with pyonephrosis, surgical indications for pyeloplasty include progressive HN, loss of renal function and the presence of symptoms during follow-up. Decreased DFR or severe HN alone at clinical presentation did not indicate surgery. 

### 2.2. Statistical analysis

Statistical analyses were performed using Statistical Package for Social Sciences (SPSS) version 22.0 (SPSS Inc., Chicago, IL, USA). The Kolmogorov–Smirnov test was performed to assess normal distribution. Parametric variables were analyzed by independent *t*-test and non-parametric variables by Mann–Whitney U test. The homogeneity of variance was determined by the Levene test. To compare qualitative variables, Chi-square test with Fisher exact test correction was used. The Pearson correlation coefficient was used to measure the strength of a linear association between two variables. Forward multiple regression was used for multivariate analysis, which included only variables that reached statistical significance in univariate analysis. The level of statistical significance was set at p < 0.05.

## 3. Results

A total of 182 (132 male, 50 female) patients with unilateral HN (122 left kidneys, 60 right kidneys) were included. During follow-up, 92 patients underwent surgical repair of UPJO and unilateral pyeloplasty was performed. Surgical indications of these patients were as follows: progressive HN in 44 patients, loss of renal function in 31 patients, and the presence of symptoms in 17 patients. The median age of patients was 9 (range: 0 – 365 days) days at the time of diagnosis of HN and 7 (range: 6 days–36 months) months at the time of the surgery. The mean postoperative follow-up period was 44.7 (12–99) months for the surgically-treated group.

According to clinical and radiologic findigs at diagnosis and during follow-up, 90 patients were treated conservatively, and these patients constituted the follow-up group. The median age of the follow-up group at the time of diagnosis of HN was 4 (range: 5 days–12 months) months. The mean follow-up period was 50.5 (range: 12–72 months) months. Comparative data of these two groups are summarized in Table 1. There was no statistically significant difference between groups in terms of age, sex, side of hydronephrosis, and duration of follow-up.

**Table 1:** Demographic, anatomic, and functional values of the patients undergoing surgical intervention and conservatively followed.

	Surgically-treated group(n = 92)	Conservativelly follow-up group(n = 90)
Median age at diagnosis	9 (0–365) days	4 (5 days–12months) months
Male/Female	3.1/1	2.9/1
Follow-up period	Mean: 44.7 (12–99) months	Mean: 50.5 (12–72) months
Side of HN	Left N: 62 (67.4%)Right N: 30 (32.6%)	Left N: 60 (67%)Right N: 30 (33%)
SFU Grade of HN (n )Grade I:Grade II:Grade III:Grade IV:	006626	056340
Initial scintigraphy (n)DMSAMAG-3	5232	66
Postoperative scintigraphy (n)DMSAMAG-3	3636	-
Contralateral compensatory hypertrophy, n (%)	31 (33.7)	0
Contralateral transient minimally HN, n (%)	18 (19.6)	0

HN: Hydronephrosis, DMSA: Dimercaptosuccinic acid scintigraphy, MAG-3: Mercaptoacetyltriglycine scintigraphy.

Contralateral transient minimal hydronephrosis was detected in 18 (9.9%) patients during follow-up. The CTMH emerged at the median age of 3 (range: 2–8 months) months and disappeared within an average of 5 (range: 2–10 months) months. Renal pelvis anterior-posterior diameters, SFU grades of these intact kidneys, bladder volume during examination, and RPAPD changes of the affected kidneys before and during this period are shown in Table 2. All these patients were in the surgically treated group (18/92, 19.6%). RPAPD in the affected kidney tended to decrease when CTMH was developed (Table 2). A mean of 7 (0–13) months after the emergence of the CTMH, the affected kidney was referred for surgery (Figure). Median age at the time of surgery was 10 (5–16) months. None of the patients in follow-up group developed CTMH during a mean duration of 50.5 months. 

**Table 2:** Ultrasonographic and scintigraphic findings of the patients with CTMH.

	RPAPD (mm)/ SFU Grade of contralateral kidney during emergence of CTMH	Bladder volume (cc) during the USG	Renal RPAPD changes of the affected kidney; before and during emergence of CTMH (mm)	Pre-op DRF of affected kidney (%)	Post-op DRF of affected kidney (%)
1	6/I	2	40–30	39	21.8
2	9/II	10	25–17	42	50
3	6/I	empty	20–17	45	43
4	8.3/II	empty	20–15	44.5	32
5	5.9/I	empty	39–30	52	40.8
6	7/I	empty	30–30	40	-
7	5/I	5	25–35	40	30
8	10/II	6	45–40	44.6	55
9	7.3/I	empty	50–15	32	30
10	10/II	empty	25–30	28	27
11	5.8/I	10	20–15	49	33.4
12	10.5/II	2.5	28–28	49	48
13	5/I	2	25–35	44.5	55
14	5.4/I	empty	25–15	39	49
15	9/II	empty	30–20	46.7	45
16	8/II	empty	30–30	42	45.4
17	9/II	empty	37–20	13	9
18	5.5/I	empty	35–25	36	18

RPAPD: Renal pelvis anterior-posterior diameter, CTMH: Contralateral transient minimally hydronephrosis, CCH: Contralateral compensatory hypertrophy, DRF:

**Figure 1:**
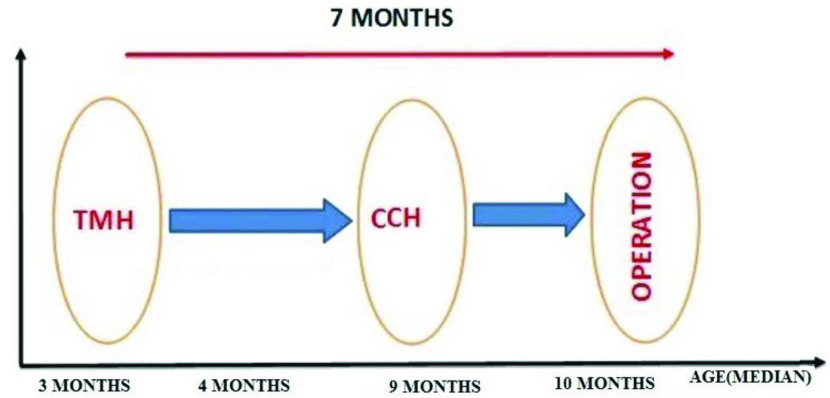
The time relationship between CCH, CTMH, and operation. TMH: Transient minimally hydronephrosis, CCH: Contralateral compensatory hypertrophy, DRF: Differential renal function.

Preoperative loss of DRF rate was 66.6% (12/18) in CTMH (+) patients, while it was 29.7% for the rest of the patients (Table 3). Despite a successful operation (no perioperative and postoperative complications, no postoperative urinary tract infection and no sign of obstruction in Tc-99m MAG-3 scintigraphy), six patients had postoperative loss of DRF (Table 2 and 3).

**Table 3:** The comparison of renal functional and ultrasonographic outcomes in patients with and without CTMH.

	Patients with CTMHn = 18	Patients without CTMHn = 74	P value
Compensatory hypertrophy, n (%)	18 (100)	13 (17.5)	0.00001
Preoperative SFU Grade, n (%)Grade III:Grade IV:	11 (61)7 (39)	55 (74)19 (26)	0.380.38
Preoperative mean DRF, %	34.6 (9–49)	45.6 (28–58)	
Preoperative decrease in DRF, n (%)	12 (67)	21 (30)	0.0049
Postoperative decrease in DRF, n (%)	6 (33)	0 (0)	0.001

CTMH: Contralateral transient minimally hydronephrosis, DRF: Differential renal function.

Contralateral compensatory hypertrophy was developed in 31 (17%) patients during follow-up. The CCH was diagnosed at the median age of 9 (range: 2–16) months. The percentile values of the contralateral kidneys at the presentation were 80 and below (5–80), while it was 95 and above (95–100) after compensation. All these patients were in surgically treated group (31/92, 33.7%) and 18 (58%) of them were CTMH-detected cases previously. The median time between CTMH detection and CCH development was 5.5 (range: 2–10) months. None of the patients in the follow-up group developed CCH during follow-up.

A univariate analysis determined the following risk factors of the deterioration in the affected kidney: CCH and CTMH. In a multiple logistic regression analysis, CTMH was found to be the only independent predictor of deterioration in the affected kidney in the infants followed up with isolated hydronephrosis and poor prognosis (p = 0.011 and p = 0.0004, respectively) (Table 4).

**Table 4:** Univariate and multivariate logistic regression analyses for the deterioration in the affected kidney in the infants followed up for isolated hydronephrosis.

Variables	Univariate analysis			Multivariate analysis		
	OR	%95 CI	P	OR	%95 CI	P
Age	1.01	1.001–1.019	0.56			
Sex	1.567	0.551–4.452	0.399			
Laterality	0.611	0.206–1.814	0.375			
Time between presentation and operation	0.984	0.956–1.014	0.302			
Preop SFU	1.692	0.628–4.563	0.299			
Preop APD	1.003	0.963–1.045	0.889			
Preop Parenchymal Thickness	1.002	0.814–1.235	0.982			
Preop DRF	0.957	0.901–1.017	0.157			
CCH	4.83	1.257–18.624	0.031			
CTMH	13.158	1.442–120.070	0.022	21.171	2.021–221.7	0.011

APD: Renal pelvis anterior-posterior diameter, DRF: Differential renal function, CCH: Contralateral compensatory hypertrophy, CTMH: Contralateral transient minimally hydronephrosis.

## 4. Discussion

Both anatomical and functional findings of affected kidneys at diagnosis and during follow-up are the main criteria for differentiating obstructive pathologies in prenatal hydronephrosis. On the other hand, obstruction does not only affect the ipsilateral kidney but it also causes significant changes in the contralateral kidney. These changes are the possible result of functional disorders in the affected kidneys, and it is a well-known condition. Hinman termed this condition as a “renal counterbalance” [9]. Renal counterbalance is the response of the contralateral kidney to the reduction of the number of functional nephrons in the affected kidney. Starting from this point of view, it seems logical to consider that various detectable changes in the contralateral kidney might be an indicator of functional changes in the affected kidney. Koff et al. pointed out that CCH can be an early sign of reduced function in the affected, hydronephrotic kidney with obstruction [6]. On the other hand, Brandell et al. reported that CCH was a common finding that might have been manifested without any functional loss in hydronephrotic kidney and should not be used as an auxiliary finding in the follow-up and management of a hydronephrotic kidney [10]. In our series, 31 of 92 patients who underwent surgery had developed CCH on contralateral kidney, while none of the patients, who were followed-up conservatively, developed CCH on serial US exams. All 31 patients with CCH were operated according to our surgical indication criteria, and UPJ obstruction was confirmed by both retrograde urography and operative findings. In addition, CCH was found to be associated with functional defects of the affected kidney (67% of patients) and, in 18 of these patients, there was loss of function preoperatively. Our study also supports the finding that CCH is associated with worsening of the affected kidney (p = 0.0017).

There were 18 patients in our series who developed contralateral minimally hydronephrosis during follow-up. The previous examinations of these kidneys did not show any sign of HN and/or calyceal enlargement, neither did the patients hydrated intravenously prior to US investigation. Moreover, all patients had at least one normal ultrasonography report after neonatal period. The median age of the patients at diagnosis of contralateral minimally hydronephrosis was 3 (range: 2–8) months. Since this USG finding was disappeared in all patients within a median 5 (range: 2–10) months period, it was named as “contralateral *transient* minimally hydronephrosis”. Although CTHM appears to be a harmless clinical finding, we have documented several clinically significant changes in both affected and contralateral kidneys associated with CTHM. Firstly, all these patients developed CCH on average 5.5 months after the onset of CTMH and CTMH regressed immediately after the development of CCH. Secondly, all patients with CTHM were underwent surgical treatment for UPJO within an average of 7 (2–13 months) months after the emergence of CTMH. Thirdly, all these patients had preoperative renal deterioration on the affected kidney. When we evaluate all these findings, it seems that there is a clinical sequence in some of unilateral HN cases. During the clinical course of these cases, CTMH develops first. Then, CCH emerges, and CTMH disappears shortly after the emergence of CCH. This process is followed by ipsilateral renal failure/loss of function and surgical treatment (Figure). 

All these changes can be explained by the effects of partial/chronic urinary obstruction on renal hemodynamics and functions. In guinea-pig models, the renal blood flow of the affected kidney can be halved due to chronic ureteral obstruction [11]. This condition leads to decrease in the number of perfused glomeruli in obstructed kidneys [12]. At the same time, renal blood flow on the contralateral kidney increases while unilateral urinary obstruction occurs. More interestingly, renal blood flow may increase even before any changes are detected in the obstructed kidney [13]. Up to 3 weeks, the number of perfused glomeruli are increased in the contralateral kidney, and this effect is the main reason of hyperfiltration of the nephrons [11]. The filtration rate of the intact/ contralateral kidney increases in proportion to the loss of function in the obstructed kidney. This means that the higher the loss of function of the diseased kidney, the higher the GFR and urine production of the contralateral kidney, which possibly causes minimally HN at the beginning and then results in compensator hypertrophy. 

Secondary HN caused by increased urine flow and higher pressure in the collecting system is well documented in the literature [14]. This condition is also seen in some cases with nephrogenic diabetes insipidus [15].

In our series, patients who developed CTMH and CCH constituted a small proportion of patients treated surgically (19.6% and 33.7%, respectively). The most important question at this point is that why all surgically-treated patients that have proved urinary obstruction did not develop CTMH and/or CCH. Since our study is retrospective, it is not possible to explain this question with our findings. But we may argue that some of CTMH cases were missed because of a short period between the appearance and disappearance of CTMH. This period can meet the period between the follow-up USGs. Another argument might be that the different severity and/or nature of UPJO may determine CTMH development. 

Among 90 patients in the follow-up group, CTMH was not detected, and all patients with CTMH were underwent surgery. This finding supports that CTMH is closely related with severe urinary obstruction. To the best of our knowledge, CMH was not previously described in the literature and can be the earliest sign of the worsening of the affected kidney. 

The retrospective nature of the study, the small number of patients with CTMH, and the comparison of different renal functions according to Tc-99m DMSA and MAG-3 data in some patients are the main limitations of this study. Our results need to be strengthened by larger series of patients and prospective studies.

In conclusion, the results of our study support the association between the deterioration of the affected kidney and contralateral transient minimal hydronephrosis. Among the variables investigeted, CTMH was found to be an independent predictor of the deterioration in the affected kidney and poor prognosis in infants who are followed-up with isolated unilateral hydronephrosis. Contralateral transient minimal hydronephrosis can predict the gradual loss of DRF on the affected kidney, and, together with clinical and other laboratory findings, it can also predict the need for surgical intervention before contralateral renal injury develops/worsens. This leads us to believe that contralateral transient minimal hydronephrosis can be considered as an “early alarm” sign for both worsening of the affected kidney and the need of surgical intervention.

## Informed consent

The ethics committee approval from Cerrahpasa Medical Faculty, İstanbul University-Cerrahpaşa was provided for this study (No: A 2016-1). No written informed consent was necessary for this type of study. The data used to support the findings of this study are available from the corresponding author upon request.
